# Visibility Graph Analysis of Heartbeat Time Series: Comparison of Young vs. Old, Healthy vs. Diseased, Rest vs. Exercise, and Sedentary vs. Active

**DOI:** 10.3390/e25040677

**Published:** 2023-04-18

**Authors:** Alejandro Muñoz-Diosdado, Éric E. Solís-Montufar, José A. Zamora-Justo

**Affiliations:** 1 Instituto Politécnico Nacional, Unidad Profesional Interdisciplinaria de Biotecnología, Mexico City 07340, Mexico; amunozdiosdado@gmail.com (A.M.-D.); eric.montufar@gmail.com (É.E.S.-M.); 2 Instituto Tecnológico de Santo Domingo (INTEC), Santo Domingo 10602, Dominican Republic

**Keywords:** visibility, tachograms, *k-M* slope, complex networks, average degree, average path length

## Abstract

Using the visibility graph algorithm (VGA), a complex network can be associated with a time series, such that the properties of the time series can be obtained by studying those of the network. Any value of the time series becomes a node of the network, and the number of other nodes that it is connected to can be quantified. The degree of connectivity of a node is positively correlated with its magnitude. The slope of the regression line is denoted by *k-M*, and, in this work, this parameter was calculated for the cardiac interbeat time series of different contrasting groups, namely: young vs. elderly; healthy subjects vs. patients with congestive heart failure (CHF); young subjects and adults at rest vs. exercising young subjects and adults; and, finally, sedentary young subjects and adults vs. active young subjects and adults. In addition, other network parameters, including the average degree and the average path length, of these time series networks were also analyzed. Significant differences were observed in the *k-M* parameter, average degree, and average path length for all analyzed groups. This methodology based on the analysis of the three mentioned parameters of complex networks has the advantage that such parameters are very easy to calculate, and it is useful to classify heartbeat time series of subjects with CHF vs. healthy subjects, and also for young vs. elderly subjects and sedentary vs. active subjects.

## 1. Introduction

Nowadays, the scientific community is studying new tools for physiological signal analysis that can be successfully used for the classification of different groups by computational algorithms [1,2,3,4]. Especially, the analyses of electrocardiograms (ECG) by different methods have shown potential applications for the prediagnosis of several diseases [5]. These tools could be helpful for physicians when giving a diagnosis. Some traditional techniques for the analysis are based on measurements of time and frequency [6,7]. For example, De Abreu et al. [8] analyzed the performance of cyclists with different kinds of training by using time and frequency domain markers on beat-to-beat heart period series. However, these methods have limitations due to the sampling frequency of the equipment used to measure and the sensitivity of the results to the definition of the filter bandwidth [9]. For instance, vagal modulation could be studied in high-frequency heartbeat time series, known as respiratory sinus arrhythmia [10]. Hence, new methodologies have emerged for the analysis of these kinds of signals. Guzzetti et al. [11] showed that signals in a group containing patients with chronic heart failure and in a healthy control group have significant differences using linear and nonlinear methods. Therefore, the measurement of the signals’ complexity and the application of nonlinear dynamic techniques have shown relevant results for classifying the signals of healthy subjects from those of diseased subjects [2,4,12]. Moreover, the influence of aging, gender, and the physical condition of subjects has been studied with heart rate variability (HRV) analysis with entropy calculations [9,13,14,15]. Furthermore, the results of Porta et al. [16] suggest that age is a factor in the decrease in vagal modulation and increase in sympathetic modulation, which reduce the complexity of cardiovascular regulation.

On the other hand, to characterize the irregular behavior of time series data, alternative techniques have recently been applied in different areas of science, such as network analysis, based on the concepts of statistical physics and graph theory [17,18]. The idea of transforming a discrete set of data into a network involves considering a set of nodes and the links between them. The transformation of a time series into a network allows the investigation of the dynamics of complex systems through analysis of the properties of the network [19,20]. Several methods have been proposed to convert time series into networks as well as to characterize these series in terms of the parameters with which the network is described. For example, Zhang and Small [21], starting from time series with periodic properties, obtained complex networks representing each cycle as a node. The work of Xu et al. [22] is also important; they analyzed the properties of complex networks and the distribution of subsets of the networks’ graphs. Gao et al. [23,24] proposed a methodology to study chaotic time series and developed a new network, which they called a multivariate recurrence network.

One of the most important methodologies in this field of research is the visibility graph algorithm (VGA) [25], which consists of transforming a time series into a complex network while maintaining its basic characteristics and making use of network theory to characterize the original data. Through this mapping, the dynamic characteristics of the time series become the characteristics of the network, and conversely, the features of the time series can be deduced from those of the network. This algorithm has recently been applied in various areas, such as finance [26,27,28,29,30], weather forecasting [31,32,33], medical science [34,35,36,37], and especially in real [20,38,39,40,41,42], experimental [19], and synthetic seismicity analyses [43].

In the VGA approach, a segment connects two time series values that can see each other. In terms of graph theory, each time series value is assigned a node, and there exists a connection (or edge) between two nodes if there is visibility between them. The degree of connectivity is the number of connections per node. Each node is visible at least to its nearest neighbors (left and right), and there is invariance under related transformations of the time series [25]. The graphs developed using the VGA method have been shown to transform periodic, random, and fractal time series into regular, random, and unscaled networks, respectively [25,44,45].

In the seismicity field, Telesca et al. [38] have used the VGA method to study sequences of seismic magnitude, and they observed power law behavior in the distribution of connectivity degrees. This unscaled signature on connectivities is concomitant with other scale-invariant properties observed in earthquake research. Earthquakes are complex phenomena, both in terms of their spatial and temporal characteristics, and many of the laws of seismology exhibit fractal properties, as reflected by scale exponents [46,47].

In the visibility graph methodology, the degree of connectivity *k* is defined for each event according to its magnitude and position in the time series. The *k-M* parameter is the slope of the straight regression line, with the magnitude on the *x*-axis and the degree of connectivity on the ordinate axis. Telesca et al. [19] conducted a similar analysis on regional seismicity in Pannonia and proposed that the correlation between these two parameters is almost universal. Similar results have been obtained when analyzing seismicity time series obtained experimentally [19]. Additional studies in this direction have been performed by Azizzadeh-Roodpish et al. [42,48] for Southern California and the Alaskan Crustal and Aleutian subduction zones; by Khoshnevis et al. [41] on the seismicity of northern Iran; and by Pérez-Oregon et al. [43] in synthetic earthquake time series. All of these studies have strengthened this universal linear correlation.

The variability in the human heart rate is the result of the combination of different physiological control systems that allow the body’s functioning to adapt to physical, environmental, or other changes. Time series of long-term R–R intervals (or tachograms) are nonlinear time series that present fractal properties. The behavior of heart rate variability is altered with age, when subjects exercise, and when they are ill [9,49,50,51,52,53]. When HRV is reduced, there is an increased risk of cardiovascular disease [54,55], and higher mortality has also been reported in patients with diseases of the circulatory system [56,57]. As tachograms are nonlinear, inhomogeneous, and non-stationary time series, nonlinear methods can better capture changes in HRV than linear methods [12,58,59,60]. To analyze these time series, many nonlinear methodologies have been used, such as detrended fluctuation analysis (DFA) [61,62,63], fractal dimension [64,65], and multifractal analysis [4,66].

Changes in HRV induced by low or intense physical activity have been widely studied [1,13,15,67,68,69,70,71,72,73,74]; when the subject is exercising, cardiovascular disorders can be detected that are not observed when the subject is inactive [70]. Therefore, by introducing extra work for the heart while exercising and monitoring the subject’s heart activity with an electrocardiograph, it may be possible to discern heart problems that are not seen when the subject is at rest [68]. Heart rate has been shown to increase during physical activity due to the withdrawal of the parasympathetic system and increased sympathetic activity (of course, this depends on the intensity of the exercise) [69]. Furthermore, it has been reported in animal models that improving physical fitness can reduce problems caused by heart disease [75].

Very interesting works analyzing cardiac interbeat series using the visibility algorithm have been published; for example, Jiang et al. [76] applied the VGA to study the dynamics of the human heartbeat during chi meditation and yoga. Their results indicated that VGA analysis can reveal changes caused by meditation training, which manifest as regular heartbeats, and they concluded that this methodology constitutes a reliable tool for the assessment of the dynamic changes in heartbeat under different physiological and pathological conditions, even when using a short series with only 400 beats. In 2018, Bhaduri et al. [3] studied heartbeat time series taken from the Physionet databases, corresponding to two groups of subjects: one healthy and the other with congestive heart failure. As the distribution degree of the nodes was found to satisfy a power law, they proposed the parameter of power of scale-freeness in visibility graph (PSVG), which could significantly differentiate between healthy and diseased subjects and also allow for the determination of different stages of the disease. Choudhary et al. [77] used the horizontal visibility algorithm and proposed the parameter of grouped horizontal visibility entropy (GHVE) to quantify the complexity as a function of the probability distribution of the observations. They concluded that the proposed GHVE measure is more accurate in distinguishing healthy from pathological subjects than its ungrouped HVE counterpart. Therefore, it is better suited for the detection of changes related to aging, disease severity, and activity levels. Other researchers [78] have investigated the possible impact of meditation on the complexity of heart rate signals using the VGA. Using data from Physionet, they analyzed the dynamics of heartbeat time series both before and during meditation by examining the complexity of the VGs using the graphical index complexity (GIC) [79]. In general, the results obtained showed that heart rate signals were more complex during meditation.

On the other hand, there are other parameters characterizing complex networks, such as the average degree, which is the mean number of neighboring nodes for each node, and the average path length, which is the mean distance between all pairs of nodes [17].

The objective of this work is to study the behavior of the *k-M* parameter for a totally different complex system: the heart rate variability time series [50]. To do this, heartbeat time series obtained from different people in various situations are studied: first, we compare healthy young and elderly subjects, then healthy subjects and patients with congestive heart failure (CHF), and, finally, subjects at rest and while exercising. In addition, we analyze the average degree and average path length of the networks obtained for each time series. We show, in this work, that these parameters are useful to determine differences in time series and, remarkably, that the selected parameters are the simplest to obtain from a network due to the low computational costs for time and resources. It is worth mentioning that the main contribution of this work is to identify the network parameters that can be implemented for the classification of the heartbeat time series of patients with CHF vs. healthy subjects, young vs. elderly subjects, and sedentary vs. active subjects. Although VGA is not a new tool, in this paper we explore its use in heartbeat time series, something that has not been widely studied, in order to enlarge the range of tools that can be used for series analysis.

The remainder of the work is organized as follows. Section 2 presents the methods of the VGA performed (Section 2.1) and the databases used for analysis (Section 2.2); this last subsection is divided according to the kinds of series considered. First, we generate white, pink, and Brownian noises to verify the differences between network parameters in these artificial series; then, we obtain series from Physionet [80] with registers of young and elderly subjects and another database with healthy subjects and subjects with CHF; and, finally, the third database was generated by our own records of subjects at rest and during exercise. Section 3 presents the results of the *k-M* slope, the average degree and average path length of all databases mentioned above, and the statistical analysis to validate significant differences between the groups. Section 4 opens the discussion and the contribution of the work. Finally, Section 5 shows the concluding remarks.

## 2. Materials and Methods

### 2.1. Visibility Algorithm

**Figure 1:**
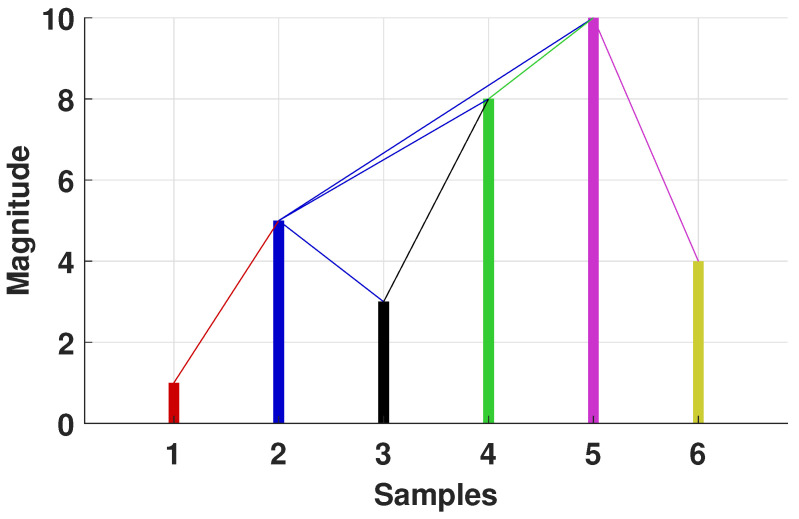
Description of the visibility method. Each bar represents the magnitude of a single value from a time series (called a node) and continuous lines represent connections between nodes. If a node with higher magnitude (taller bar) is between two non-adjacent nodes, these other nodes do not have a connection.

The visibility graph algorithm is a method that maps a time series into a complex network. We illustrate the method in Figure 1 in terms of a bar graph, where the height of the bars represents the magnitude of the events. Two events are connected if they are visible to each other, and this happens if any event between them intersects the straight line that connects them. This visibility algorithm [25] has attracted a lot of attention as it can reveal the nonlinear characteristics of time series. By mapping the time series into a graph with connected nodes, this complex graph (or network) inherits various properties of the time series; for example, random time series result in random graphs, periodic time series result in random graphs, and time series with fractal properties result in scale-free networks [17,18]. Let x(i),1≤i≤N be a time series composed of *N* data, and consider each data point as a node in the VGA. Nodes *x*(*i*) and *x*(*j*) are connected only when the following condition is satisfied [25]:

(1) $$\forall l\in \left(i,j\right);x\left(l\right)<x\left(i\right)-\left(j-l\right)\frac{x(j)-x(i)}{j-1}.$$

This is the basic definition of an undirected visibility graph. The following properties always hold for a VG graph [25]:

- A node is visible at least to its nearest neighbors (left and right).
- The edges do not have a direction.
- Visibility is not affected by the scaling of either horizontal or vertical axes, nor horizontal or vertical translations.

There are various modified versions of the method, such as the horizontal visibility algorithm and the directed visibility algorithm, in which the connection is defined in only one direction. The direction can be forward (away from the node) or backward (toward the node) [81,82,83]. In some applications, these variations have been applied with good results; for example, in the analysis of chaotic periodic signals [81,82], as well as in other randomly correlated ones, for which the correlation [81] or the reversibility have been characterized [83].

We can build matrices for the visibility graph of a time series, called the adjacency matrix. The dimension of the adjacency matrix is the number of nodes in the network. If there is a connection between the nodes corresponding to a certain row and column, the element of the matrix is equal to one; otherwise, it is equal to zero. For an undirected visibility graph, this matrix is symmetric, with the elements of the main diagonal equal to zero. There are usually ones around the main diagonal, as neighboring events are more likely to be visible to each other. In the case of a directed visibility graph, the matrix is asymmetric. The degree of connectivity of each node is obtained as the sum of the number of connections in the matrix for each corresponding row [25].

The reliability of this methodology has been confirmed through its application to both artificial fractal series and real series [25]. As has been reported, this method can provide good results with relatively small series; for example, Jiang et al. [76] have claimed that reliable results can be obtained with only 400 data.

In seismicity, the term *k-M* refers to a graph in which the degree of connectivity *k* is plotted against the magnitude of the earthquake; however, for any time series, the magnitude refers to the time series value that corresponds to that node. It is expected that a small value of the time series will have a small value of *k*, and a large value of the time series (which, in the visibility diagram, looks like a very high bar) would have a high degree of connectivity as, from this point, many other bars can be seen [84].

### 2.2. *k-M* Slope, Average Degree, and Average Path 0 for White, Pink, and Brownian Noise

We illustrate the *k-M* calculations for 100 time series generated using white, pink (1/f), and Brownian noise. Self-affine series were obtained with *N* = 1024 data and with values of the spectral exponent β as follows: β=0.5 (white), β=1 (pink), and β=2 (Brownian). The methodology of [85,86,87] was used to generate the time series. In addition, the average degree and average path length of networks generated from the aforementioned series were also calculated. Furthermore, analysis of variance (ANOVA) was used to find significant differences between the networks’ parameters calculated; if this was found, the contrast test of Fisher’s least significant difference (LSD) was performed to determine exactly which groups showed statistical differences.

### 2.3. Heartbeat Time Series

#### 2.3.1. Young and Elderly Healthy Subjects

In order to evaluate the complexity lost with age [9], the VGA was applied to heartbeat time series obtained from Physionet [49,80], a database containing data from 20 elderly (68–85 years old) and 20 young (21–34 years old) healthy subjects, whose electrocardiography signals (ECG) were recorded while they remained in a resting state watching the movie Fantasia. Each subgroup included equal numbers of women and men. We extracted samples with 1500 events from each time series. Then, we obtained the visibility graph and the corresponding complex network for each time series, and the *k-M* value was obtained by fitting the connectivity degree versus the magnitude (i.e., the duration of the time interval in seconds) of each R–R interval. In addition, the average degree and average path length were also calculated. In order to prove that the differences between the obtained values were statistically significant, we performed Student’s t tests with a significance level of 0.05 for comparison.

#### 2.3.2. Healthy Subjects and CHF Patients

In addition, heartbeat time series of healthy subjects and patients with congestive heart failure (CHF) were obtained from Physionet [80], and we considered the following data:

- 54 registers over 24 h from subjects with normal sinus rhythm R–R interval (30 men, aged 28.5–76; 26 women, aged 58–73).
- 29 registers over 24 h from subjects with CHF, New York Heart Association (NYHA) classifications I, II, and III.
- 15 registers over 24 h from subjects with severe CHF, NYHA classifications III and IV (11 men, aged 22–71; 4 women, aged 54–63).

We obtained six-hour sub-series when the subjects were sleeping and also six-hour sub-series when patients were awake. However, for comparison purposes, we only separated sub-series with 1500 data, as with the data on healthy old and young people from the previous section. To obtain the *k-M*, average degree, and average path length, the same procedure was followed as in the previous section. A Student’s *t* test was also performed with a significance level of 0.05 for the comparison of these values calculated for both groups. With this analysis, we intended to verify if there is also a loss of complexity in the signals of subjects with CHF, as occurs in the aging process, which is associated with an increase in sympathetic modulation due to the constant cardiac stress generated by the disease.

#### 2.3.3. Cardiac Interbeat Series Obtained at Rest and While Exercising

In total, tachograms of 152 subjects at rest and exercising (walking on a treadmill) were analyzed. Of these 152, 84 were young men and women with an average age of 20.8 years, while the other 68 were middle-aged adults with a mean age of 51.9 years. The measurements were repeated for all subjects. For each participating subject, personal information was collected, including age, gender, weight, height, and so on. Subjects with any disease related to the cardiovascular system were discarded, and it was ensured that they were not taking medication of any kind. The tests were carried out in the morning, at a suitable temperature. The average body mass indices of the subjects were as follows: for young people, it was 25.24 ± 3.53; for middle-aged adults, it was 28.08 ± 4.54. For each record, there were two measurements: a 60 min rest period and a 30 min walking period on a treadmill. A total of 4096 and 2048 data were analyzed for resting and exercise series, respectively. At the beginning of the test, seven electrodes were placed on the subjects to take their ECG recordings, using a Fukuda Denshi Holter monitor model FM-180 with a sampling frequency of 125 Hz. Once the 60 min rest period had ended, the young subjects (under 30 years of age) were put on a commercial electric treadmill to walk at 1.56 m/s for 30 min. The speed for the older subjects was 1.34 m/s. The beat-by-beat signal, or tachogram, was obtained for each digitized ECG using a peak detection algorithm.

The participants were volunteers and signed the informed consent form. The protocol for this research was approved by the Ethics Committee of the FES-UNAM-Mexico under document number CE/FESI/022020/1348. Before the test, all subjects answered the International Physical Activity Questionnaire (IPAQ) in order to classify their physical activity [88]. With the help of this questionnaire, the subjects were classified into three levels of physical activity (high, moderate, and low); all comparisons were made with subjects with high and low physical activity because time series of subjects with moderate results were discarded, so comparisons were made with 31 series of active middle-aged adults, 24 series of sedentary middle-aged adults, 51 series of active young subjects, and 30 series of sedentary young subjects.

In addition, all visibility graphs were generated from the R–R series of each person in the previously described databases, allowing us to compare the *k-M*, the average degree, and the average path length for the different populations. In order to prove that the differences between the obtained values were statistically significant, we performed a hypothesis test by ANOVA followed by Fisher’s LSD contrast test to determine which groups showed significant differences. The objective of this analysis was to identify changes in series through VGA. Subjects who performed regular exercise achieved the inhibition of the sympathetic system during physical activity, causing an improvement in the vagal response [8], which could be seen in minimal changes between series taken at rest and those during exercise. This fact was evaluated in both young and middle-aged adult subjects.

## 3. Results

### 3.1. Visibility Parameters from White, Pink, and Brownian Noise

**Figure 2:**
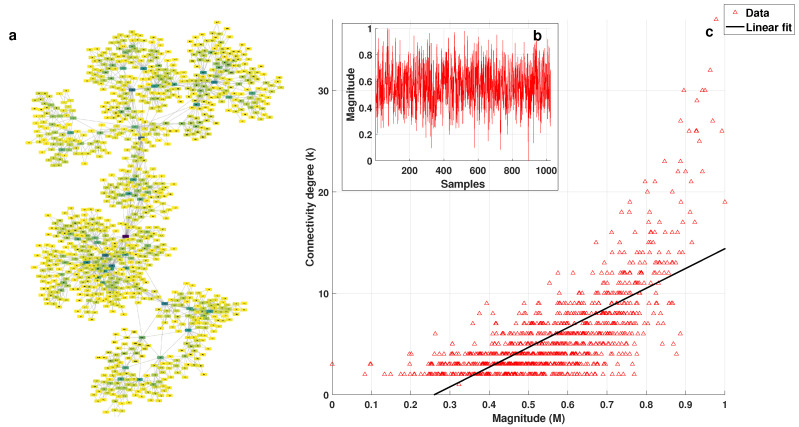
(**a**) Visibility graph generated for the white noise shown in (**b**); and (**c**) *k* vs. *M* plot with linear fit.

**Figure 3:**
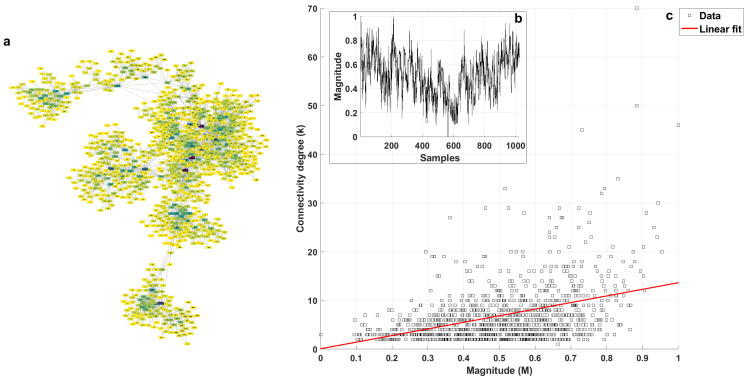
(**a**) Visibility graph generated for the pink noise shown in (**b**); and (**c**) *k* vs. *M* plot with linear fit.

**Figure 4:**
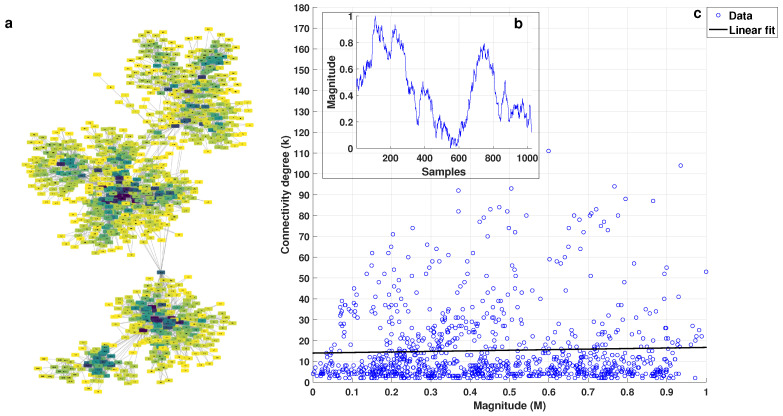
(**a**) Visibility graph generated for the Brownian noise shown in (**b**); and (**c**) *k* vs. *M* plot with linear fit.

Figure 2a shows the visibility graph obtained for white noise, where the node color depends on its degree (k), with darker colors representing higher degrees. The series that generated this graph is shown in Figure 2b. Figure 2c shows the plot of connectivity degree (*k*) vs. magnitude (*M*) for each value from the series, and the linear fit of the data by mean least squares, which was used to obtain the *k-M* slope, can also be observed. Figure 3 corresponds to the *1/f* noise, while Figure 4 corresponds to the Brownian noise. It can be seen that the appearance of the networks, as these types of noise are different, presented the following characteristics:

- White noise has more dispersion between nodes, providing a lower average degree in the network and a greater average path length.
- With Brownian noise, the nodes form many large clusters with high connectivity, leading to a greater average degree and a lower average path length.
- For pink noise, the average path length and average degree values were between those obtained for white and Brownian noise.

From the plots of *k* (connectivity degree) vs. *M* (magnitude), we obtained the linear fits: for white noise, *k* = 4.8787 M+10.4871; for 1/f noise, *k* = 13.5515 M+0.0853; and, for Brownian noise, *k* = 19.4626 M−5.0672.

In the graph of *k* vs. *M*, despite the fact that the distribution of points was not close to a straight line, we proved that the correlation was significant with the corresponding hypothesis test for the white, *1/f*, and Brownian noises. Considering a level of significance of 0.05, we obtained *p*-values less than $3.5\;\times \;10^{-3}$ in all cases; therefore, the correlation was significant despite the large spread of the data in the *k* versus *M* plots, and other mathematical models were not considered to fit the data since the linear fit was statistically validated, as indicated above.

On the other hand, 100 different series of white noise with 1024 data as well as 100 series of 1/f noise and Brownian noise were generated. For each of the three hundred series, the value of the slope *k-M* was obtained. The averages for each type were as follows: for white noise, 21.6203 ± 2.1061; for 1/f noise, 15.4359 ± 2.4233; and, for Brownian noise, 5.8246 ± 6.9253.

**Figure 5:**
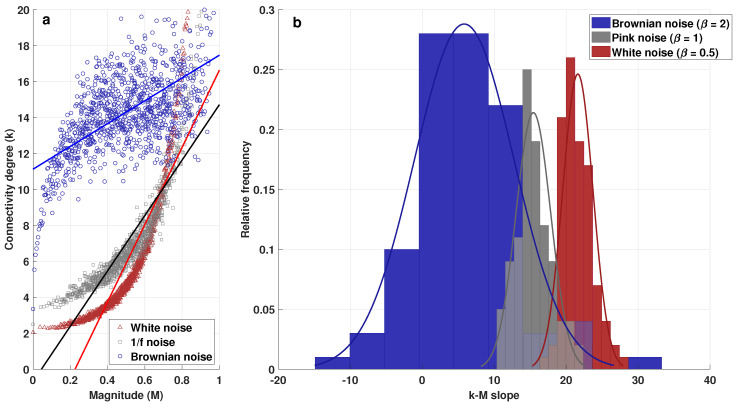
(**a**) Average connectivity degree vs. average magnitude plot for white, pink, and Brownian noise; and (**b**) histogram and distribution of *k-M* slopes from 100 series of the above-mentioned noises.

Figure 5a shows the average values of *k* and *M* from the 100 series for each kind of noise. It is worth mentioning, in this context, that the hypothesis tests to validate the correlation coefficients of the linear fit to calculate the *k-M* slope were also performed. In addition, Figure 5b shows the distribution of the *k-M* slopes obtained from the series, from which the differences between the *k-M* values of these three kinds of series can be observed. The average values of the means are clearly separated, although the values of the standard deviations are large, especially for Brownian noise. However, the ANOVA results indicated significant differences between the *k-M* of the three groups. Since differences were found by ANOVA, posthoc tests were necessary for evaluating pair-wise differences; hence, Fisher’s least significant difference (LSD) procedure was performed, and the results showed that there were significant differences between all the pairs of noise types ($p\;<\;1.0\;\times \;10^{-5}$ in all cases, α = 0.05).

It is precisely the very different values obtained for the average of the *k-M* slopes that motivated us to think that this parameter may distinguish between different time series, especially those of cardiac interbeat. In terms of the β spectral exponent, it has already been reported that the R–R series has a β value of around one or a DFA exponent value of one [60,61,62], which corresponds to the *1/f* noise; however, deviations from this value may occur in the elderly or in people with heart disease.

**Figure 6:**
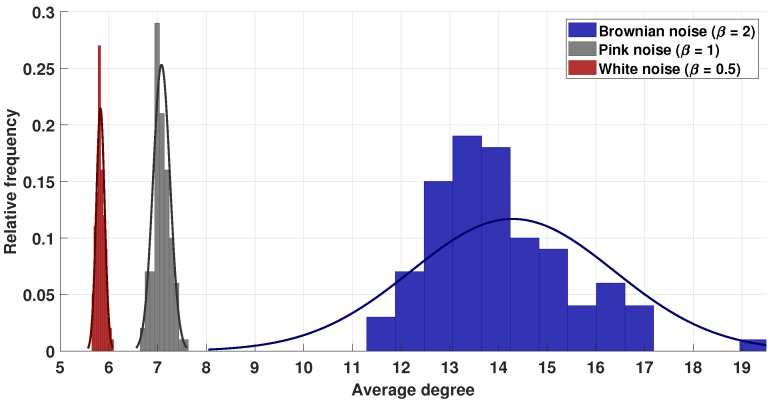
Histogram of average degree for white, pink, and Brownian noises.

In addition, we compared the average degree and average path length in the white, pink, and Brownian noise networks. Figure 6 shows the histogram of the average degrees, from which we observed similar differences to those mentioned above. The values obtained were as follows: 5.8249±0.0855 for white noise, 7.0797±0.1734 for pink noise, and 14.2925±2.0838 for Brownian noise. Furthermore, an ANOVA was performed, and the results indicated significant differences between the average degrees for the three series. Thus, a contrast pair-wise test was necessary, and in all cases, there were significant differences when the corresponding Fisher’s LSD test was performed ($p<1.0\times 10^{-5}$ in all comparisons). On the contrary, the average path length values did not present significant differences.

### 3.2. Analysis of Physionet Database

By analyzing the Fantasia database, it was possible to observe a difference between the *k-M* plots of the two populations. While the mean and standard deviation of the slopes of the *k-M* plots for young people had lower values, these values were higher for the older subjects. The obtained values for the mean and standard deviation for both populations can be observed in Table 1. These two populations were statistically different, with a *p*-value of p=0.004. It is worth mentioning that the average degree and average path length calculated for these series can also be observed in Table 1; however, these results did not indicate significant differences between populations. These results suggest that the *k-M* value could be a network parameter that is useful to evaluate the aging process and the complexity loss presented in the series of the elderly subjects, which also indicates a reduction in regulatory mechanisms.

Similar results were observed when comparing the healthy subjects with the congestive heart failure (CHF) patients from the Physionet database. The series were split into asleep and awake periods, which were compared separately for the two populations. In both periods, the healthy subjects reported lower values for the slope in the *k-M* plot than the CHF patients, and these differences resulted in significant *p*-values ($p_{awake}=9.0\times 10^{-5}$, $p_{sleep}=9.0\times 10^{-5}$). The results of this analysis are provided in Table 1. Furthermore, Table 1 also presents the average degree and average path length calculated for these series, from which it was found that there were significant differences between the average degree of healthy and diseased subjects in awake and asleep period ($p_{awake}=2.7\times 10^{-3}$, $p_{asleep}=2.4\times 10^{-4}$); meanwhile, for the average path length, only significant differences were observed for the asleep series ($p_{asleep}=3.73\times 10^{-4}$). Hence, these results indicate that these network parameters can be useful tools for the classification of tachograms from healthy subjects and those with CHF, but apparently it is better to make the comparisons when the patients are asleep.

### 3.3. Heartbeat Time Series of Exercising Subjects

**Figure 7:**
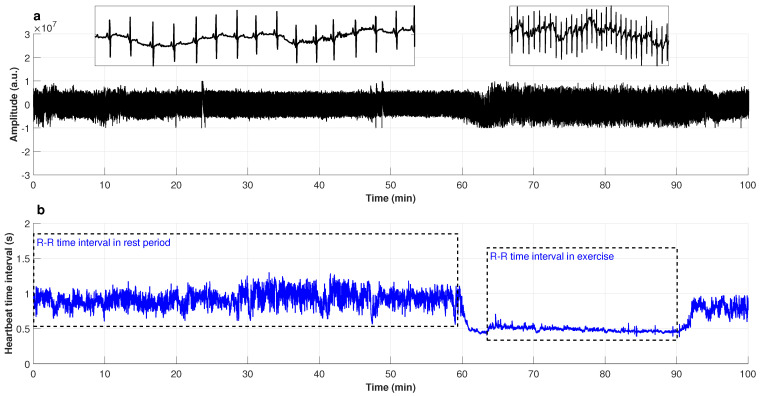
(**a**) Electrocardiography signal; and (**b**) R–R time interval series in rest period and during physical activity.

Figure 7 shows the recorded ECG signal from a young subject during rest and exercise testing. From the Figure, it can be easily identified when the person is at rest and when they are on the treadmill due to the time intervals between the R peaks decreasing in the exercise period, caused by the blood and oxygen demand of the skeletal muscle cells to perform the movement. Therefore, it is easy to differentiate the tachograms at rest and those of physical activity once the artifacts are removed. When the Holter Fukuda Denshi FM 180 was used to measure the ECG signal of subjects at rest and walking at low speeds, the signal obtained was very stable and has almost no artifacts, so our results were not affected by the presence of many non-stationarities [89]. The time series for physical activity were not taken from the time the subject got on the treadmill, as the signal takes time to stabilize; that is, data in the rest–exercise transition region were not considered.

**Figure 8:**
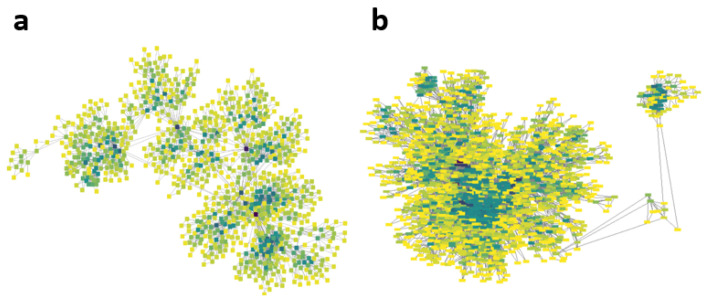
(**a**) Visibility graph of a young subject at rest; (**b**) visibility graph of same subject during exercise. The node color depends on its connectivity degree, where darker colors represent higher degrees.

A sample of networks for rest and activity for a young subject are shown in Figure 8, from which it can be observed that the rest series graph generated more clusters and the nodes have a greater connectivity degree compared with the graph derived from the exercise series. We compared the *k-M* values for young and middle-aged subjects’ time series at rest and during physical activity, from which it was found that differences were higher than those in the previous results. The *k-M* mean and standard deviation for this test are displayed in Table 2. In this case, an ANOVA was used to compare the calculations of the *k-M* slope for the four groups. Using this test, significant differences were found between the *k-M* means. Hence, we proceeded to performed a contrast test by using Fisher’s LSD method, obtaining the following results:

- Series recorded of young subjects and middle aged adults at rest did not show significant differences (p=0.26).
- Series of young subjects at rest comparing with those during exercise showed significant differences ($p<1\times 10^{-5}$).
- Series of middle-aged adults at rest comparing with those recorded while exercising showed significant differences ($p<1\times 10^{-5}$).
- Series of young subjects and middle-aged adults during exercise showed significant difference ($p=5.7\times 10^{-3}$).

These results showed that the *k-M* slope of the two populations at rest are similar and this is attributed to the classification of all subjects as healthy, since it has been reported [13] that healthy subjects, during rest periods, conserve their heart rate variability and tachogram complexity. However, during physical efforts, the HRV and heart interbeat time series’ complexity decreased, producing *k-M* slopes that were significantly higher in the exercise series than those during rest in both populations.

In order to ensure that the observed results were trustworthy and reproducible, we compared the *k-M* values of young and adult subjects from the Fantasia database with those we measured using a Holter monitor. As the Fantasia series were recorded while the subjects were watching a movie, we compared these series with our subjects at rest. The means and standard deviations can be observed in Table 1 and Table 2 for the Fantasia database and the subjects at rest, respectively. The Student’s t tests indicated that there were no significant differences for these populations; that is, young subjects from the Fantasia database were comparable to those measured at rest (and the same for the adults). The *p*-values obtained in these statistical tests were $p_{young}=0.56$ and $p_{adult}=0.81$.

**Figure 9:**
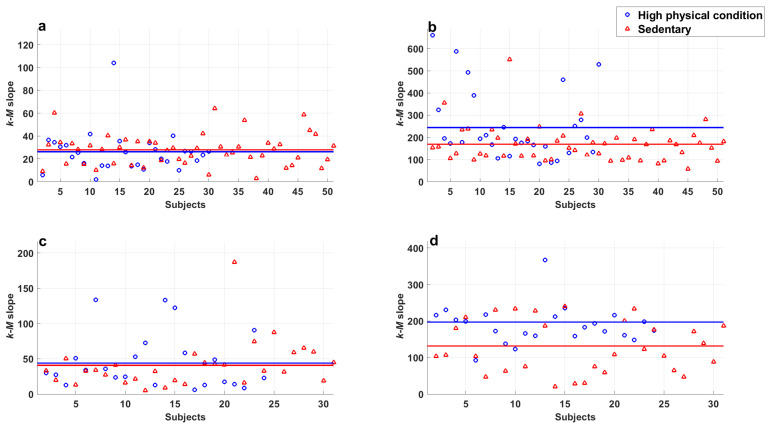
Comparison of *k-M* slope from sedentary young subjects and high-physical activity young subjects in (**a**) resting period and (**b**) during exercise; and those corresponding to middle-aged subjects in (**c**) resting period and (**d**) during physical activity. Markers represent every value of *k-M*, and continuous lines indicate the means.

The IPAQ results were used to compare the *k-M* slope differences between subjects with high physical activity levels and those who preferred a sedentary lifestyle. The *k-M* mean and standard deviation of these groups at rest and during activity can be observed in Table 3. In addition, Figure 9 depicts the *k-M* values obtained from series for the two groups, with the continuous line representing the average. The ANOVA was performed and the results showed significant differences. Furthermore, the following results were obtained by the Fisher’s LSD test:

- There were no significant differences between series of subjects with high physical activity levels and sedentary subjects in both populations at rest (p>0.36 in all cases).
- There were significant differences found between series of subjects with high physical activity levels and sedentary subjects in both populations at rest and those recorded during exercise ($p<1.3\times 10^{-5}$ in all cases).
- A significant difference was found in series recorded during exercise of young subjects with high physical activity levels and sedentary subjects ($p=9.7\times 10^{-6}$).
- A significant difference was found in series recorded during exercise of middle-aged adults with high physical activity levels and sedentary subjects ($p=9.7\times 10^{-4}$).
- Series of young subjects who exercise regularly and sedentary middle-aged adults during exercise did not show a significant difference (p=0.12).

It can be observed in Table 3, that the *k-M* slopes obtained for the series of subjects increase during exercise, above all in series of young subjects the increase is higher compared with that of middle-aged adults. In addition, the *k-M* is greater in subjects with a sedentary lifestyle in both groups. This fact produces an overlap in the series of sedentary adults and young subjects with high physical activity levels.

It is worth mentioning that significant differences in this parameter were not obtained for the resting series. We also attributed this to all subjects having been declared as healthy; during the rest period, heart rate variability (HRV) is conserved, while during heart effort, the variability decreases. These results indicate that the hearts of subjects with high physical activity levels manage activity conditions better and maintain HRV, while the opposite is observed in sedentary individuals.

Furthermore, the average degree of networks obtained from rest and exercise time series were analyzed. Table 2 shows the results of the means and standard deviations for young subjects and adults. In this case, the average degree of exercise series decreased in physical activity. Significant differences between rest and physical activity periods were obtained by ANOVA test and the contrast Fisher’s test showed the following:

- Average degree values of series recorded of subjects of both groups at rest showed significant differences (p=0.01).
- Average degree values of young subjects’ series at rest compared with those during exercise showed significant differences ($p<1\times 10^{-5}$).
- Average degree values of middle-aged adults’ series at rest compared with those recorded while exercising also showed significant differences ($p<1\times 10^{-5}$).
- Series of young subjects and middle aged adults during exercise did not show a significant difference (p=0.16).

**Figure 10:**
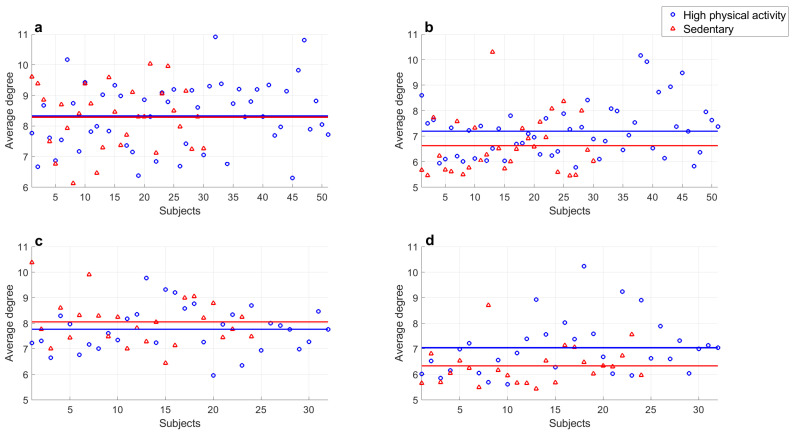
Comparison of average degree from visibility graph of sedentary young subjects and high-physical activity young subjects in (**a**) resting period and (**b**) during physical activity; and those corresponding to middle-aged subjects in (**c**) resting period and (**d**) during physical activity. Markers represent every value of average degree and continuous lines indicate the means.

Similar to the *k-M* slope, comparisons of the average degree calculated from subjects with high physical activity levels and sedentary subjects were performed, with the averages and standard deviations shown in Table 3 and a graphic representation in Figure 10. The ANOVA results indicated that there were significant differences between the averages of the eight groups, and the following results were found by Fisher’s LSD test:

- Series of young subjects with high physical activity levels and sedentary subjects at rest did not show a significant difference (p=0.85).
- Series of middle-aged adults with high physical activity levels and sedentary subjects at rest did not show a significant difference (p=0.30).
- Significant differences were found between the average degree values of series of subjects with high physical activity levels and sedentary subjects from both populations at rest and those recorded during exercise ($p<6.0\times 10^{-3}$ in all cases).
- A significant difference was found in exercise series of young subjects with high and low physical activity levels ($p=1.5\times 10^{-2}$).
- A significant difference was found in exercise series of middle-aged subjects with high and low physical activity levels ($p=1.0\times 10^{-2}$).
- Series of young subjects and middle-aged adults classified with high physical activity levels while exercising did not show a significant difference (p=0.50).
- Series of sedentary young subjects and middle-aged adults in exercise period did not show a significant difference (p=0.28).

It was also found that there were no significant differences in the rest series; again, this was attributed to all subjects in database being healthy, such that the real difference in HRV was observed only during physical activity. In addition, the results suggest that the average degree calculated in series of subjects who exercise regularly are similar regardless of age, and the same occurs for sedentary subjects.

The average path length of networks obtained from resting and exercise series were also analyzed. Table 2 shows the results in terms of the averages and standard deviations of young subjects and adults in both conditions. In this case, the average path length is lower during exercise and this difference is greater in middle-aged adults. The ANOVA was performed and the Fisher’s LSD test showed the following:

- Average path length values of series of both groups at rest did not show significant differences (p=0.22).
- Average path length values of series during rest period and exercise of both groups showed significant differences ($p=6.0\times 10^{-3}$ in all cases).
- Average path length values of series of both groups recorded during exercise did not show significant differences (p=0.99).

**Figure 11:**
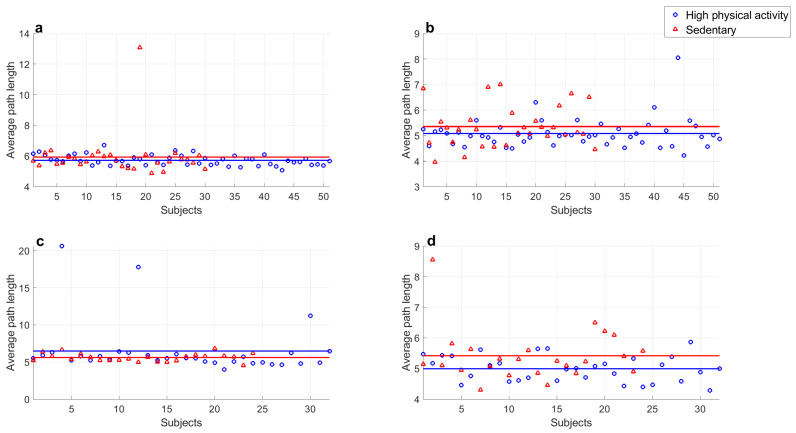
Comparison of average path length from visibility graph of sedentary young subjects and high-physical activity young subjects in (**a**) resting period and (**b**) during exercise; and those corresponding to middle-aged subjects in (**c**) resting period and (**d**) during physical activity. Markers represent every value of average path length and continuous lines indicate means.

Finally, the comparisons of the average path length of networks from subjects with high and low physical activity were performed. The results are provided in Table 3 and depicted in Figure 11, and we obtained the following significant differences:

- Average path length values of middle-aged adults with high physical activity levels at rest and during exercise showed a significant difference ($p=4.5\times 10^{-5}$).
- Average path length values of young subjects with high physical activity levels at rest and during exercise showed a significant difference (p=0.021).

In these comparisons, significant differences were only obtained in the average path length of series of active subjects at rest and during physical activity. On the contrary, this parameter for series of sedentary subjects in both populations was similar.

## 4. Discussion

Once we had analyzed the selected visibility graph parameters for white, pink, and Brownian noises and we had verified the differences, we decided to applied these calculations in heartbeat time series, since it has been reported that series of young and healthy subjects present long-term correlations [1], such as in the pink noise. However, some conditions (age or illness) modify this behavior, causing decorrelations or short-term correlations in series, such as in the white and Brownian noises, respectively [60,61,62,90].

Generally the work was divided in three stages. In the first stage, we analyzed the series of young and elderly subjects watching the movie Fantasia, and significant differences were obtained in the *k-M* values between both groups, which were attributed to a complexity loss due to age [14,66]. This reduction also indicated alterations in the homeostatic regulatory mechanisms, which may compromise the capacity of physiological adaptations before several external conditions [9,10]. The second stage showed significant differences in three parameters calculated in series of healthy and CHF subjects, especially during sleeping periods. This was attributed to the fact that the complexity changes caused by the aging process are also present in the disease process [9]. In addition, Guzzetii et al. [11] reported that neuroendrocrine activation present in chronic heart failure can also be associated with the parameters of the time and frequency domain analysis of heartbeat time series. In this work, we confirmed that these changes also could be identified with VG parameters. Finally, the third stage consisted of the comparison between series at rest and during exercise of both sedentary and high physical activity subjects. The analysis concluded that there were significant differences in three parameters mentioned above between groups in both conditions, which is associated to the fact that the subjects who regularly performed aerobic exercise promote the inhibition of sympathetic regulation and the enhancement of vagal regulation [8], which causes a reduction in the loss of complexity of the signal in athletes compared to sedentary subjects. This was also identified by the VGA realized in this work.

In this study, we showed that the value of the slope for the *k-M* plots of the visibility graph, the average degree, and the average path length created from the R–R time series vary significantly for subjects under different conditions. It was confirmed that *k-M* values are useful for classifying heartbeat time series of young and elderly subjects due to these shown significant differences. In addition, with these parameters it can be distinguished between series of healthy subjects and patients with CHF, series of subjects at rest and during exercise, and series of sedentary subjects and subjects with high physical activity levels. Furthermore, the networks parameters selected are some of the simplest and fastest to calculate and, even so, the results were effective in identifying between different groups. This methodology has the advantage over traditional techniques of not being sensitive to the definition of the filter bandwidth to analyze the series.

Several studies [1,2,4,8,12,13,51,52,53] have showed the correlation between the results of nonlinear dynamic techniques applied to heartbeat time interval series (or tachograms) and the health status or age of a subject. When we observed the graphical representation of the networks obtained (Figure 2a, Figure 3a, Figure 4a and Figure 8), noticeable differences could be observed with the naked eye. However, the task of distinguishing between different types of time series should not be based solely on visual inspection. For this reason, several parameters were considered; in particular, in this work, we emphasized the parameter *k-M*, which has been used with great success in the analysis of seismic time series [38]. As we have attempted to show, the three network parameters used can distinguish between cardiac interbeat time series of young subjects and the elderly, between healthy subjects and CHF patients, people at rest and people exercising, as well as sedentary people and people who exercise regularly. The latter allowed us to glimpse some possible applications to help characterize the physical state of people, as the complex networks obtained using the VGA algorithm from the cardiac interbeat time series of sedentary people are very different to those of people who exercise regularly, as has already been reported according to other types of parameters [13,72].

Therefore, our work adds complementary information to the results that other authors have reported, with the advantage that they were applied to a wider variety of groups of subjects. As mentioned above, the VG algorithm has been used in several previously reported analyses [3,76,77,78], in which the time series of heartbeats were studied. However, the parameters that have been used to study them previously differed from those used in this work; in fact, in no previous work has the *k-M*, the average degree, and the average length of the path been used together to distinguish heartbeat time series from subjects under different conditions. Hence, this is the first time that the VGA algorithm has been used to analyze heartbeat time series of subjects who exercise in an attempt to distinguish between sedentary and active individuals. This means that these parameters can be used to characterize the physical condition of a person, and may be used to measure the progress of people who undergo an exercise routine to improve their physical condition.

As has been reported by Bhaduri et al. [3], this methodology can distinguish between healthy individuals and individuals with congestive heart failure. We also considered this aspect, but using the three parameters mentioned above, we found that we can also distinguish between the series of heartbeats of young and old people. Of course, such separations are made with respect to the mean values, namely, the means of the two populations are different and their difference is statistically significant, but this does not mean that there are no overlaps; that is, at the time of performing the separation, there will always be some cases of healthy people who appear ill and some ill people who appear healthy. This overlap is not exclusive to our work, but also occurs in other works already published. For this reason, the joint use of several parameters is necessary to make classifications; in addition to the list of already existing ones, we propose these three relatively new parameters. In this sense, what we propose is that, if we wish for these types of tools to be used, for example, to provide a diagnosis, we should use many of these techniques together (even considering traditional analysis techniques), including the ranges of values for these and other parameters with respect to the control groups of healthy people, in such a way that deviations from these values can be quantified. Then, when several deviations are observed in different parameters, it can be affirmed that the person has a health concern, which could help the physician to provide a final diagnosis.

## 5. Conclusions

In this work we used the visibility algorithm to map time series in complex networks and calculate for these networks the parameters *k-M*, average degree, and average path length, initially for white, pink, and Brownian noise time series. We used a broad set of parameters that are used for complex networks, but we decided to only focus on those that are easier to evaluate and that also gave us better results. Since the *k-M* parameter and the average degree of the aforementioned noise networks showed significant differences, we concluded that such parameters can be used to distinguish between heartbeat time series from different groups, for which comparisons were made, for example, in young people vs. elderly people, healthy subjects vs. CHF patients, resting subjects vs. exercising subjects, and sedentary youth or adults vs. regularly exercising youth or adults.

Using time series obtained from Physionet [80], we found that there are significant differences in *k-M* values between the heartbeat times series of young people and those of the elderly, which is attributed to the fact that heart rate variability decreases with age [66]. In addition, with Physionet data, we found statistically significant differences in these parameters when comparing series of healthy subjects and patients with congestive heart failure. In our research group, we have made electrocardiography (ECG) recordings using Holter monitors of people who are first at rest and then performing physical activity. The physical condition of the participating subjects was characterized using the IPAQ questionnaire [88], so we were able to compare heartbeat time series of people with a sedentary lifestyle (low physical activity) and people who exercise regularly (high physical activity). There are significant differences in both sedentary and active young subjects and middle-aged adults. In addition, it was found that there are no significant differences in the series of the rest period, neither in young people nor in middle-aged adults. However, during physical effort, the *k-M* slope, the average degree, and the average path length of the series from sedentary and highly physically active young and middle-aged adults showed statistically significant differences.

The *k-M* parameter has given excellent results applied to real and synthetic seismicity time series. In this case, the results showed that the calculation of this parameter with the average degree and average path length, despite them having been described several years earlier for different applications, could be useful as a new tool for ECG analysis and the identification of subjects with CHF and sedentary subjects and for evaluation of aging process. We are continuing to work on increasing the size of our database with the aim that in the future other complex network parameters can be incorporated into this methodology.

## Figures and Tables

**Table 1:** Values of mean and standard deviation for the slope of the *k-M* plots, average degree, and average path length for the Physionet database.

	*k-M* Slope	Average Degree	Average Path Length
Young (Fantasia)	37.00 ± 4.05	7.48 ± 0.88	5.09 ± 0.26
Elderly (Fantasia)	65.00 ± 6.40	7.90 ± 1.28	5.02 ± 0.43
Healthy (awake)	47.38 ± 6.12	9.15 ± 1.35	4.62 ± 0.63
CHF (awake)	137.73 ± 11.58	8.15 ± 1.23	4.37 ± 0.81
Healthy (asleep)	48.09 ± 4.88	8.41 ± 1.26	4.79 ± 0.55
CHF (asleep)	120.97 ± 10.07	7.68 ± 0.97	4.37 ± 0.46

**Table 2:** Mean and standard deviation of the slope of *k-M* plots, average degree, and average path length for young and middle-aged subjects at rest and during exercise.

	*k-M* Slope	Average Degree	Average Path Length
Young (rest)	27.24 ± 14.14	8.32 ± 1.07	5.80 ± 0.89
Adults (rest)	50.15 ± 20.04	7.87 ± 0.98	6.41 ± 3.47
Young (exercise)	196.97 ± 118.60	6.99 ± 1.10	5.18 ± 0.68
Adults (exercise)	214.27 ± 50.34	6.28 ± 0.75	5.36 ± 0.87

**Table 3:** Values of mean and standard deviation for the slope of the *k-M* plots, average degree, and average path length of young and middle-aged subjects classified as subjects with high physical activity levels and subjects with sedentary lifestyle, in resting period and during physical activity.

Condition	Subjects	*k-M* Slope	Average Degree	Average Path Length
**Rest**	Young subjects with high physical activity levels	27.83 ± 13.53	8.33 ± 1.09	5.72 ± 0.34
	Young subjects with sedentary lifestyle	26.23 ± 17.99	8.28 ± 1.043	5.93 ± 1.40
**Exercise**	Young subjects with high physical activity levels	169.23 ± 81.84	7.20 ± 1.04	5.08 ± 0.59
	Young subjects with sedentary lifestyle	244.13 ± 151.22	6.63 ± 1.13	5.35 ± 0.80
**Rest**	Adults with high physical activity levels	40.82 ± 33.59	7.76 ± 0.89	6.48 ± 3.62
	Adults with sedentary lifestyle	44.07 ± 39.35	8.05 ± 0.92	5.61 ± 0.55
**Exercise**	Adults with high physical activity levels	131.53 ± 71.64	7.04 ± 1.12	5.00 ± 0.43
	Adults with sedentary lifestyle	196.96 ± 65.71	6.33 ± 0.75	5.423 ± 0.85

## Data Availability

All data used in the present work can be requested from the corresponding author.

## Abbreviations

The following abbreviations are used in this manuscript:

ANOVA	Analysis of Variance
CHF	Congestive heart failure
DFA	Detrended fluctuation analysis
ECG	Electrocardiogram
GHVE	Grouped horizontal visibility entropy
GIC	Graphical index complexity
HRV	Heart rate variability
HVE	Horizontal visibility entropy
IPAQ	International Physical Activity Questionnaire
LSD	Least significant difference
NYHA	New York Heart Association
PSVG	Power of scale-freeness in visibility graph
VG	Visibility graph
VGA	Visibility graph algorithm
